# Radiocapitellar joint plasty for missed monteggia fracture with radial head deformity in children: a retrospective study

**DOI:** 10.3389/fped.2024.1374224

**Published:** 2024-07-09

**Authors:** Xuedong Li, Huiling Tian, Kun Lu, Xiaobo Jing

**Affiliations:** ^1^ Pediatric Orthopaedic, Zhengzhou Orthopaedic Hospital, Zhengzhou, Henan, China; ^2^ School of Pharmacy, South-Central Minzu University, Wuhan, Hubei, China

**Keywords:** missed monteggia fracture, articular cartilage, radiocapitellar joint plasty, dysplastic radial head, osteoarthritis

## Abstract

**Purpose:**

The retrospective study reviewed the clinical and radiological outcomes of patients treated with radiocapitellar joint plasty.

**Methods:**

10 children with missed Monteggia fracture (MMF) were reviewed. The average time from injury to operation was 20 months. The average age of children who underwent the operation was 10.5 years. 6 flat and 4 domed radial heads were included. 7 type I and 3 type III MMF were identified based on the Bado classification. All children with MMF were treated by open radial head reduction with radiocapitellar joint plasty and ulnar osteotomy (UO).

**Results:**

The average union time was 4.9 ± 2.6 months. The average osteotomy angle to reduce the radial head was 15.7 ± 3.5°, and the average lengthening of the ulna was 8.2 ± 3.2 mm. The average preoperative flexion range of motion was 110.5 ± 9.1°, and the postoperative flexion range of motion was 138.8 ± 15.1° (*p* < 0.05). The average preoperative extension range of motion was 10.1 ± 3.2°, and the postoperative extension range of motion was 5.5 ± 3.3° (*p* < 0.05). The average preoperative pronation range of motion was 78.8 ± 8.7°, while the postoperative pronation range of motion was 81.1 ± 5.6° (*p* > 0.05). The average preoperative supination range of motion was 68.3 ± 9.7°, and the postoperative supination range of motion was 80.1 ± 7.8° (*p* < 0.05). The preoperative Kim score was 66.5 ± 10.9°, and the postoperative Kim score was 88.1 ± 12.6 (*p* < 0.05). The radial head was completely reduced in 9 patients, and subluxation in 1 patient. Osteoarthritis of the radiocapitellar joint was observed in 2 patients.

**Conclusions:**

Radiocapitellar joint plasty is effective surgical intervention for MMF with radial head deformity. It yields favorable functional outcomes while ensuring continued radial head reduction.

## Introduction

Giovanni Battista Monteggia first described Monteggia fracture in 1,814 and reported two cases of ulnar shaft fractures accompanied by anterior radial head dislocation (1, 2). Tardy displacement of the radial head and missed diagnosis are the main reasons for this missed Monteggia fracture (MMF) (3). Recent studies suggest that the dividing line for MMF should be over four weeks after injury, as malunion of the ulna has already occurred (1, 4). Normally, the radial head is concave in axial cross-section, with the ellipse's long axis perpendicular to the ulnar radial notch in supination (5). Kim demonstrated that these dysplastic changes may present as early as 3 months after radial-head dislocation (6). In pediatric patients, early-developed ossification centers were observed at the injured radial epiphysis, indicating that the trauma stimulated bone growth (7). The capitellum loosed its normal contour, demonstrating a flattened or convex appearance (8).

Bell Tawse initially reported a technique for surgical treatment for MMF in 1,965, which included radial head open reduction, ulna osteotomy (UO), and annular ligament reconstruction (ALR) (9). It is unknown whether dysmorphic features of the radial head are a contraindication for surgery. However, if the radial head is found to be dome-shaped and loses all its concavity, surgery of Bell Tawse tends to be unsuccessful, and the unmatched radiocapitellar joint becomes painful and may eventually develop osteoarthritis (8). In cases where elbow complaints persist after bone maturity, alternative surgery options include regular follow-up and consideration of radial head excision. It is essential to note that long-term studies have demonstrated potential risks, such as instability of the elbow and arthralgia associated with radial head resection in MMF (10, 11). However, this procedure is commonly considered as salvage surgery and is not encouraged.

Arthroplasty can restore the bony anatomy of the radiocapitellar joint and avoid re-dislocation of MMF with radial head deformity. Few reports have described the details regarding solving radial head deformity techniques, and no long-term investigations of clinical or radiographic results in many patients have been published.

We had performed radiocapitellar joint plasty for MMF with radial head deformity in children. This retrospective study aims to review the clinical and radiological outcomes of patients treated with radiocapitellar joint plasty.

## Materials and methods

Retrospective study subjects were identified according to inclusion and exclusion criteria.

Inclusion criteria: (1) Age 5–15 years and meet the diagnostic criteria of MMF: failure to diagnose acute Monteggia Fracture and proximal radial dislocation persists for more than 4 weeks; (2) The appearance of the radial head was classified into two types: “flat”, and “domed” according to the method of Kunihiro (12), and were confirmed by at least two experienced imaging physicians; (3) Patients signed informed consent; (4) Patients were treated by open radial head reduction with radiocapitellar joint plasty. Excluded patients: (1) Patients with Idiopathic anterior dislocation; (2) Patients with a history of elbow joint surgery; (3) Lost to follow-up; (4) Patients with concave radial head morphologic feature, pathologic fracture, open fracture, morphology of the radial head and patients with congenital radial head dislocation.

**Figure 1:**
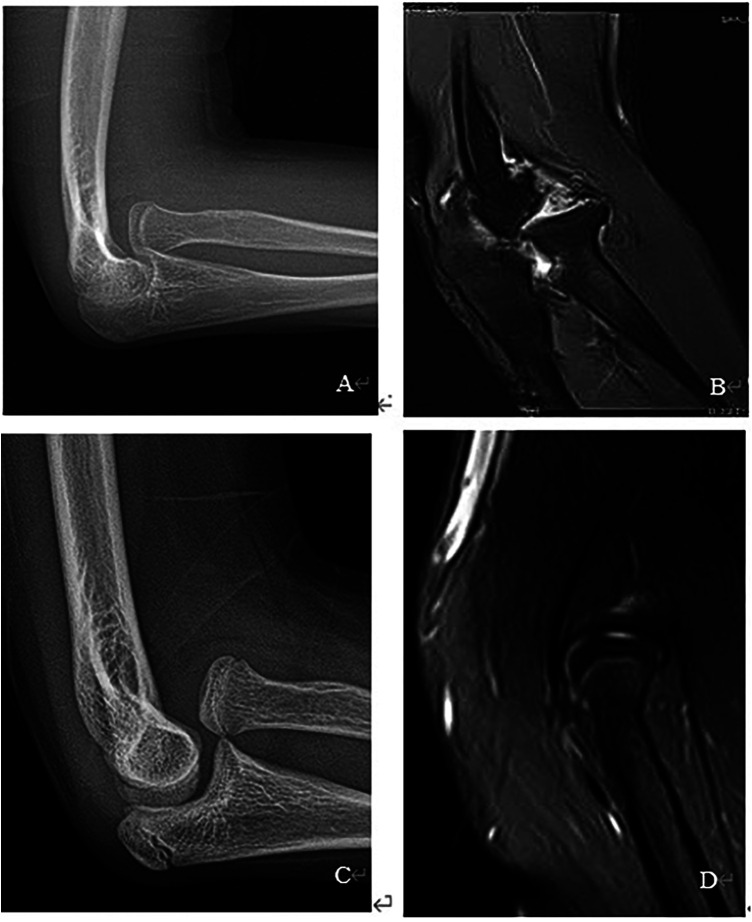
(**A**) Flat radial head in X, (**B**) flat radial head in MRI, (**C**) domed radial head in X, (**D**) domed radial head in MRI.

Between Jan 2016 and Jan 2018, a total of 10 patients were included in this retrospective study. All children with MMF were treated by open radial head reduction with radiocapitellar joint plasty and UO. Medical records of these patients were reviewed. Patients' complaints included a bulging mass, pain, and limited elbow flexion. The average time from injury to operation was 20 months (12–30 months). All patients had routine anteroposterior and lateral radiographs of the elbow, which were checked for deformity of the radial head and osteoarthritis. There are 6 flat and 4 domed radial heads (Figure 1). A classification system of the Monteggia fracture was used according to the Bado classification (13). There are 7 Type I and 3 Type III (Table 1). This study was approved by the Medical Research Ethics Board of Zhengzhou Orthopaedic Hospital.

**Table 1:** Summary of characteristics of the patients.

Characteristics	Amount
Male	8
Female	2
Age at surgery(year)	10.5 ± 2.5
Delay time to surgery(month)	20.4 ± 4.5
Follow-up time(month)	66.2 ± 4.9
FLAT	6
DOMED	4
BADO type I	7
Bado type III	3

### Surgical technique

**Figure 2:**
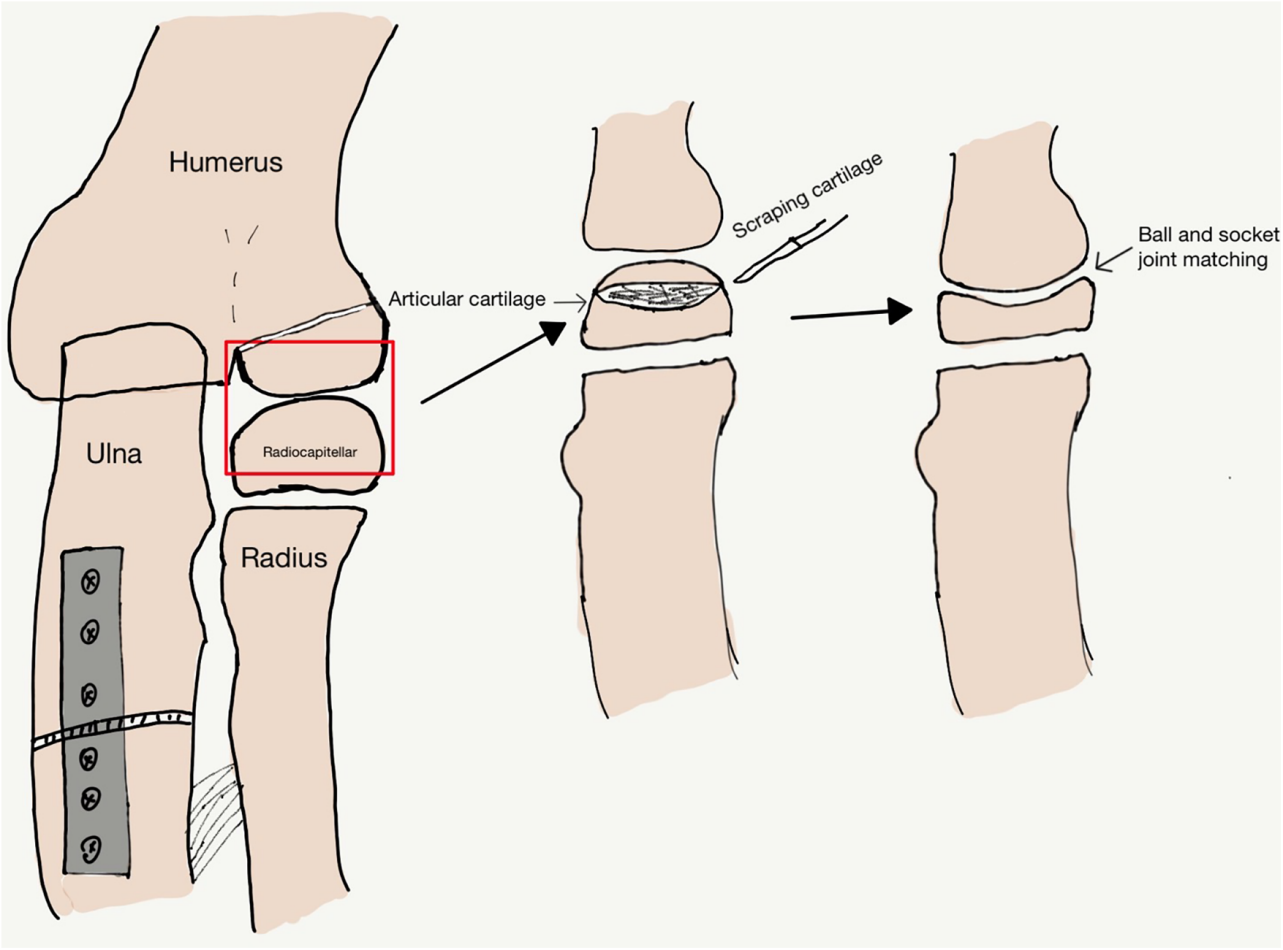
Schematic diagram of scraping off cartilage.

**Figure 3:**
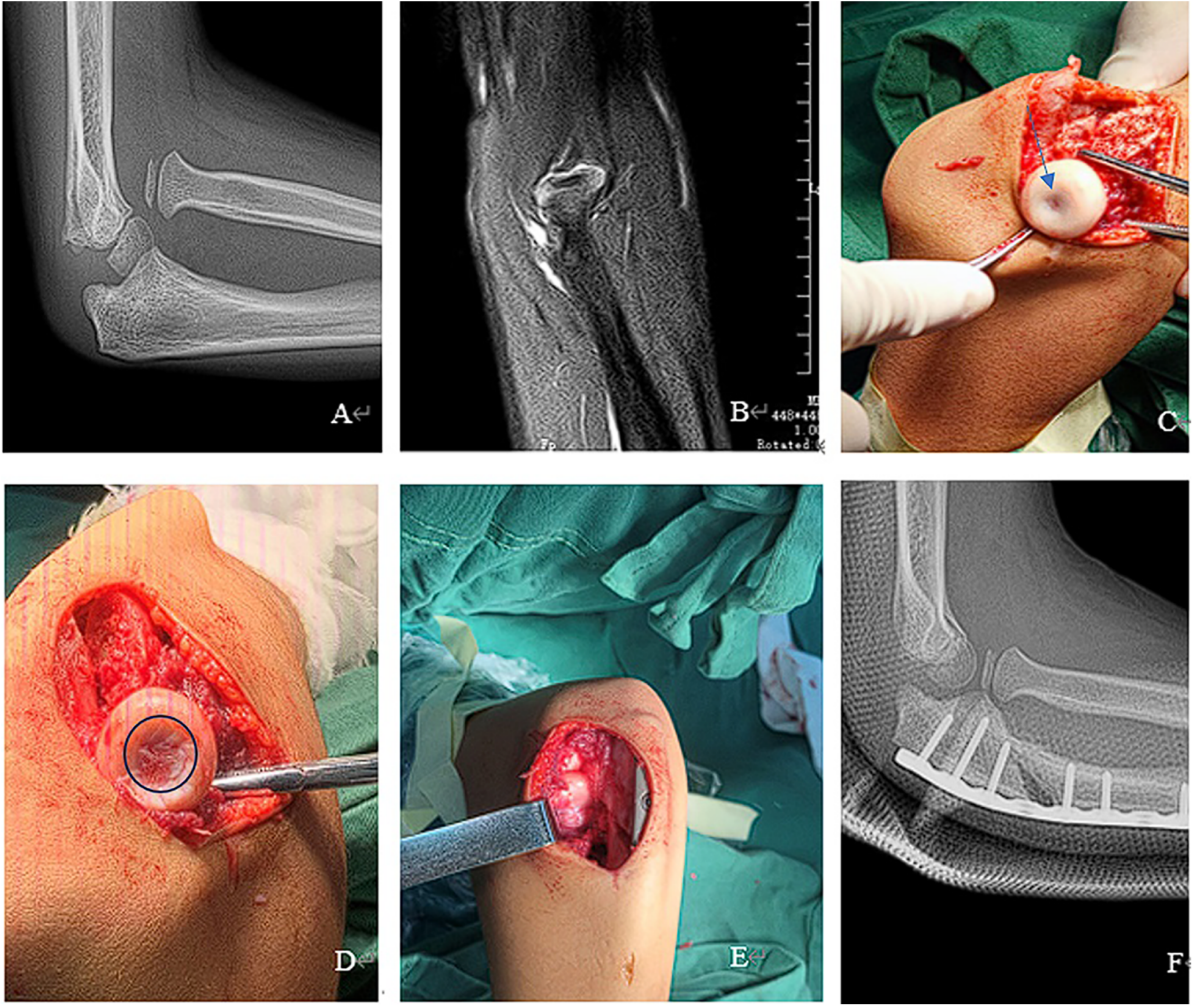
(**A**) flat radial head in X, (**B**) flat radial head in MRI, (**C**) flat radial head in operative, arrows display the area that needs to be scraped off, (**D**) scraping concave surface, circular display shows the area after scraping, (**E**) radiocapitellar joint plasty, (**F**) post-operative in X.

Under general anesthesia, patients were placed in a supine position. Surgery was performed using Bell Tawse technique and radiocapitellar joint plasty in Type I and Type III MMF. All patients were treated using the Boyd posterolateral elbow approach that exposes the radiocapitellar joint, identifies and removes the remnants of the annular ligament and fibrosis that might impede the radial head reduction. The open reduction of radial head, fibrous scar resection, ulnar angulation, and lengthening were performed. The radial head was unstable due to the shallow radial head, and joint plasty was performed at the radial head articular surface. A bistoury was used to scrape a concave surface into the articular cartilage (AC) area of the radial head to gain ball and socket structure according to the experience of the surgeon, scrape off the central area of the cartilage while preserving the edge area of the cartilage, multiple attempts, which resulted in a better match to the capitulum humerus (Figures 2,3). Try not to damage the subchondral matrix as much as possible, achieving the concave surface of radial head matches the convex surface of capitulum humerus, which could avoid subluxation of the radial head. Ulnar osteotomy was performed at the proximal ulna. The proximal part of the ulna was exposed through a 6–8 cm long incision on the ulnar side, and the wedge osteotomy was performed 4–5 cm below the ulnar olecranon where UO should allow insertion of at least two screws at the proximal part. Correction with medial convexity and elongation of the ulna for lateral dislocation of the radial head is possible, resulting in a better match to the radial notch and restoring stability of the proximal radioulnar joint (PRUJ) (14). A 5–6-hole reconstruction plate was bent to stabilize the osteotomy. In surgery, we removed only the interposed soft tissues of the radiocapitellar joint, and the remnants of the annular ligament are often attenuated and difficult to reconstruct.

Angulation of UO was based on the reduction stability identified directly and under fluoroscopy by rotating the forearm from pronation to supination and moving the elbow from full flexion to extension (Supplementary Video S1 and S2). The success of the operation is defined as no dislocation of the reduced radial head when the elbow was flexed and rotated during the operation. Osteotomy was fixed with a pre-curved plate. Decisions regarding the necessity of requiring bone grafts were made according to the surgeon's experience.

### Postoperative management

The elbow joint was immobilized in a flexion position and forearm neutral position after surgery. x-ray of the elbow was performed one week after surgery. If the reduction status of the radial head was subluxated, the plaster was used to fix the forearm in the supination position. Approximately six weeks after the operation, the anteroposterior and lateral x-ray resulting from the elbow joint were reviewed, and the plaster cast was removed. Active rehabilitation was encouraged. Periodic re-examination was conducted to monitor the possible occurrence of radiolocation and functional recovery. x-ray was taken every two months within two years, and every six months outside two years; MRI was taken every six months within two years, and every twelve months outside two years.

### Clinical evaluation

The functional outcome was assessed for all patients by the same rehabilitation specialist. At the final follow-up, the functional result was evaluated by determining the range of motion in terms of elbow flexion, elbow extension, forearm supination, and forearm pronation with a goniometer. The functional outcome of the elbow was assessed using the Kim score, which was based on deformity, pain, range of motion, and function and was interpreted as excellent (≥90), good (75–89 points), fair (60–74 points), and poor (<60 points) (15).

### Radiographic evaluation

Standard anteroposterior and lateral radiographs of the elbow and MRI were performed preoperative and at follow-up. Two surgeons evaluated the imaging material to determine.1. The final reduction status of the radial head, which was assessed according to radiocapitellar subluxation grading system: Concentric (grade 1), subluxation (Grade 2, 3, 4), dislocation (grade 5) (16). 2. Whether osteoarthritis was present, which was evaluated according to the standard proposed by Kellgren and Lawrence in x-ray, (including osteophyte formation, narrowing of joint spaces, synovitis, altered shape), and according to MRI(including joint damage that involves cartilage) (17, 18). 3. Bony union, which was defined as x-ray shows blurred bone fracture lines and continuous callus passing through the fracture line (19).

### Statistical analysis

Data analysis was performed using SPSS statistical software version 24 for Windows 7 (SPSS Inc, Chicago, USA). Continuous variable was presented as means ± standard deviations. The paired *t*-test was used to compare the continuous variables. All tests were two-sided, and *P* < 0.05 was considered statistically significant.

## Results

All patients were older than eight years during surgery and were followed up for more than 60 months. The average age of children who underwent the operation was 10.5 years old (range 8–15 years old), and the average follow-up time was 66 months (range 60–71 months). No patients experienced neural injury. K-wire was not used to reduce the radial head. Bone graft was used in 2 cases. One case occurred incision infection and was cured after dressing change. The average union time was 4.9 ± 2.6 months. The average osteotomy angle to reduce the radial head was 15.7 ± 3.5°, and the average lengthening of the ulna was 8.2 ± 3.2 mm (Table 2).

**Table 2:** Summary of clinical evaluation.

Characteristics	Pre-operative	Post-operative	P
Flexion(°)	110.5 ± 9.1	138.8 ± 15.1	0.002 < 0.05
Extension (°)	10.1 ± 3.2	5.5 ± 3.3	0.035 < 0.05
Pronation (°)	78.8 ± 8.7	81.1 ± 5.6	0.565 > 0.05
Supination (°)	68.3 ± 9.7	80.1 ± 7.8	0.012 < 0.05
Kim score	66.5 ± 10.9	88.1 ± 12.6	0.001 < 0.05

The average follow-up time was 66.2 ± 4.9 months. At the final follow-up, the average preoperative flexion range of motion was 110.5 ± 9.1°, and the postoperative range was 138.8 ± 15.1°(*p* < 0.05). The average preoperative extension range of motion was 10.1 ± 3.2°, and the postoperative range was 5.5 ± 3.3°(*p* < 0.05). The average preoperative pronation range of motion was 78.8 ± 8.7° and the postoperative range was 81.1 ± 5.6°(*p* > 0.05). The average preoperative supination range of motion was 68.3 ± 9.7°, and the postoperative range was 80.1 ± 7.8°(*p* < 0.05). The preoperative Kim score range was 66.5 ± 10.9, and the postoperative Kim score range was 88.1 ± 12.6 (*p* < 0.05) (Table 3).

**Table 3:** Summary of radiographic evaluation.

Characteristics	Amount
K-wire	0
Bone graft	2
Incision infection	1
Bone graft	5/16
Union time(month)	4.9 ± 2.6
Osteotomy angle(°)	15.7 ± 3.5
Lengthening of the ulna (°)	8.2 ± 3.2
Cubitus valgus	0
subluxation	1
Radiocapitellar osteoarthritis	2
PRUJ osteoarthritis	0

**Figure 4:**
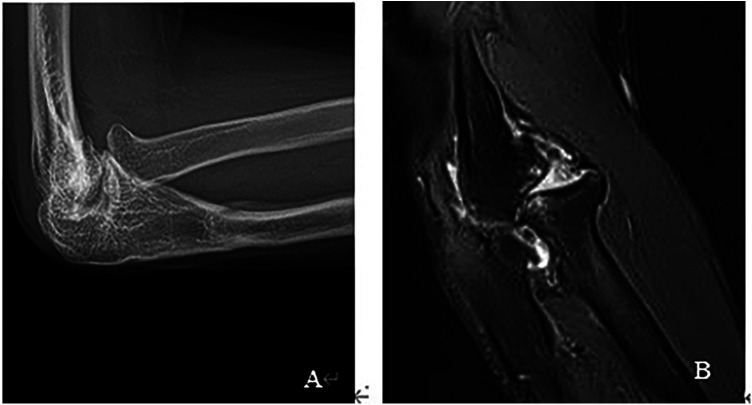
(**A**) 1 case subluxation and osteoarthritis in x-ray, (**B**) 1 case subluxation and osteoarthritis in MRI.

The radial head was completely reduced in all patients when the plaster cast was removed. There were no cases of postoperative cubitus valgus deformity in the present study. one patient occurred subluxated during elbow extension with forearm pronation which the radial head center was shifted slightly anteriorly from the center of the capitellum in 28 months postoperative follow-up, which developed osteoarthritis in 40 months postoperative on MRI and x-ray images (Figure 4). There were no occurrences of PRUJ osteoarthritis. Osteoarthritis of the radiocapitellar joint was observed in two patients. The mean age of these two patients at open reduction was 13.5 years, and the mean interval between the injury and open reduction was 26.5 months. The parents opted for regular rehabilitation training, resulting in significant improvement in pain control.

## Discussion

In our study, the difference between our method and the traditional method of Bell Tawse was the addition of radiocapitellar joint plasty surgical procedures based on Bell Tawse. Bell Tawse procedure was commonly considered as salvage surgery in the treatment of MMF with deformed radial head malformations, which was the reason the cases with traditional way of Bell Tawse were not included in this research as a comparison group. Radiocapitellar joint plasty was adopted in the current study, reducing re-dislocation incidence, pain and elbow deformity, and avoidable radial head excision and osteoarthritis.

The data from our study also suggested that radiocapitellar joint plasty for MMF with radial head deformity was generally successful. In the report by Nakamura, utilizing the original Bell Tawse technique, seven patients with the interval between the injury and open reduction longer than three years had a radial head deformity, with six patients subsequently showing subluxation of the radial head or osteoarthritis of the elbow joint (20). Shu reported that the preoperative appearance of the radial notch/head determined the surgical procedure. When the radial head and radial notch appeared concave, the rate of radial head subluxation/re-dislocation was 5.56%, lower than 81.82% that of the flat/domed radial head (21). Roger Austin reported tardy palsy of the radial nerve from MMF, our patient had no nerve damage either before or after surgery (22). Takaaki reported that six patients had dome-shaped radial heads, two showed subluxation of the radial heads, and one client experienced osteoarthritis in the elbow joint (23). In our study, one patient appeared to have radial head subluxation, and two patients experienced osteoarthritis involving domed radial head change in the radiocapitellar joint. The occurrence rate of radial head subluxation and osteoarthritis were lower than described above studies. No studies have used the radiocapitellar joint plasty method for the solution of the radial head. Hence, the current study appears to be an exploratory procedure encompassing the avoidable radial head excision. Fortunately, outcomes of the current method suggested it was effective, and no severe complications occurred.

Radiocapitellar joint plasty does not increase the risk of osteoarthritis. In Takaaki's study, two patients underwent notchplasty, with one patient experiencing the subluxation of the radial head and another showing osteoarthritis. However, he did not specify where the osteoarthritis occurred (23). Hirayama reported the importance of radial notch in achieving optimal position and avoiding excessive pressure on the radial head after the operation (14). However, Chen considered it difficult to achieve the desirable congruity and pressure because of inequable dysplastic change in the radiocapitellar and radioulnar joints (11). In our study, correction with medial convexity and elongation of the ulna achieved tension release. An adjustment might be required during surgery according to the stability of the radiocapitellar joint, which results in a better match to the radial head and PRUJ, and hopefully remodeling. The amount of radiocapitellar joint plasty was determined intra-operatively depending on matching radiocapitellar, which can easily predict the amount of correction necessary according to the preoperative x-ray and MRI. Notably, joint plasty cannot break the cartilage base, and the technique was also easily reproducible.

Restoring the stability of the bone structure is the key to treating MMF with radial head deformity. Bhaskar stressed the importance of alignment of the ulna in restoring radial head stability (24). Hyperplasia of the radial head tends to increase the instability of the elbow joint, and the stability of the elbow joint is maintained by the bony structure and joint congruity (14). With the loss of the radiocapitellar articulation, the elbow experiences a decrease in its valgus stability by approximately 33%, and this can result in patients demonstrating instability and an increase in the valgus carrying angle of the elbow (25). Radiocapitellar joint plasty can restore ball and socket joint structure, which results in a firmer grip and a larger contact surface (26).

Delay time to surgery is the main factor of radial head deformity, which contributes to consistent enlargement of the radial head and a tendency for early closure of the proximal radial physis (12). The extent of radial head deformity may be more important to prognosis rather than the actual interval from injury. Shu reported that no patient with delay time to surgery less than 6 months showed flat/domed radial head, and these final radiographic and clinical result was satisfied (21). Lu mentioned that if delay time to surgery was more than 6 months, the radial head tended to enlarge or overgrow (27). This study found that three of ten patients had radial head domed deformity, with the time between injury and corrective surgery exceeding 24 months, during the operation, the traditional method could not achieve satisfactory radiocapitellar joint reduction. AC and synovium develop from the same pool of precursors with similar gene profiles, and synovium-derived chondrocytes have stable chondrogenic activity and infer using synovium as a new cell source for AC repair (28). For AC, children seem to have a higher tolerance, and the synovium of the joints is strong in childhood. Koichi Nakamura suggested that one could expect satisfactory long-term clinical and radiographic outcomes after open reduction of MMF when the patient was less than twelve years of age or the procedure was performed within three years after the injury, and the radial head in children over twelve years of age tended to lose the capability of remodeling itself (20). Age at surgery is an important indicator for judging the growth potential of AC and synovium. In two patients with osteoarthritis, the mean age at open reduction was 13.5 years, and mean delay time to surgery was 22.4 months. The age of operation and the delay time to surgery might be the reason for worse functional and radiologic results. The study result reminded us that joint plasty seemed to have partial potential for remodeling in patients younger than 13 years. One patient occurred subluxated, and two patients showed osteoarthritis with occasional slight pain during strenuous activities. Forced reduction with radiocapitellar joint plasty may have increased stress on the radial head, or normal kinematics of the elbow joint may not have been completely reconstructed by our techniques.

The current study was subject to several limitations. Firstly, being a retrospective study, it inherently possesses some drawbacks. Secondly, the sample size was relatively small, restricting the extent of conclusions that can be drawn. A more extensive series would be beneficial in confirming the effectiveness of the method. Finally, there was limited follow-up time available for analysis.

In summary, the surgical approach for MMF in pediatric patients in this study involved open radioulnar debridement, corrective UO, radiocapitellar joint plasty, reduction of the radial head, and rigid plate fixation. This strategy serves as a valuable adjunctive method in addressing this challenging issue with favorable functional outcomes while ensuring the maintenance of radial head reduction.

## Data Availability

The raw data supporting the conclusions of this article will be made available by the authors, without undue reservation.
